# Mining phenotypes for gene function prediction

**DOI:** 10.1186/1471-2105-9-136

**Published:** 2008-03-03

**Authors:** Philip Groth, Bertram Weiss, Hans-Dieter Pohlenz, Ulf Leser

**Affiliations:** 1 Research Laboratories of Bayer Schering Pharma AG, Berlin, Germany; 2 Knowledge Management in Bioinformatics, Humboldt University, Berlin, Germany

## Abstract

**Background:**

Health and disease of organisms are reflected in their phenotypes. Often, a genetic component to a disease is discovered only after clearly defining its phenotype. In the past years, many technologies to systematically generate phenotypes in a high-throughput manner, such as RNA interference or gene knock-out, have been developed and used to decipher functions for genes. However, there have been relatively few efforts to make use of phenotype data beyond the single genotype-phenotype relationships.

**Results:**

We present results on a study where we use a large set of phenotype data – in textual form – to predict gene annotation. To this end, we use text clustering to group genes based on their phenotype descriptions. We show that these clusters correlate well with several indicators for biological coherence in gene groups, such as functional annotations from the Gene Ontology (GO) and protein-protein interactions. We exploit these clusters for predicting gene function by carrying over annotations from well-annotated genes to other, less-characterized genes in the same cluster. For a subset of groups selected by applying objective criteria, we can predict GO-term annotations from the biological process sub-ontology with up to 72.6% precision and 16.7% recall, as evaluated by cross-validation. We manually verified some of these clusters and found them to exhibit high biological coherence, e.g. a group containing all available antennal Drosophila odorant receptors despite inconsistent GO-annotations.

**Conclusion:**

The intrinsic nature of phenotypes to visibly reflect genetic activity underlines their usefulness in inferring new gene functions. Thus, systematically analyzing these data on a large scale offers many possibilities for inferring functional annotation of genes. We show that text clustering can play an important role in this process.

## Background

Phenotype descriptions are valuable information right at the interface of medicine and biology. Their main value lies in helping to dissect the relationships between diseases and genes. Therefore, large genetic screens, especially in organisms like *Drosophila melanogaster *or *Caenorhabditis elegans*, have been carried out to systematically research the phenotypes associated to genes [1-5]. Since high-throughput screening methods, such as RNA interference (RNAi), have become available also for mammals [6,7], phenotype screening has become an acknowledged and widely used component of functional genomics.

One particular problem with analyzing phenotypes is the lack of a common vocabulary to describe them. Instead, researchers use either home-grown vocabularies or plain English text. Due to the resulting heterogeneity in descriptions, automatically analyzing phenotypes is a daunting and yet relatively unexplored task. Adding to this problem, the term 'phenotype' in itself is used for a broad variety of concepts, including the descriptions of clinical diseases, the characterization of naturally occurring mutants or experimentally generated mutants, and RNAi screens or gene knock-out experiments, and sometimes even large-scale microarray gene expression data, which makes an integrated analysis of phenotypes from different experiments and laboratories particularly hard [8]. Another issue is that until very recently, no comprehensive set of phenotypes with associated genes were available. This issue has been partly addressed by the creation of phenotype databases, such as PhenomicDB [9,10] or PhenoGO [11] (see [8] for a survey on available phenotype data sets).

Probably due to these difficulties, there exist relatively few studies dealing with the analysis of phenotypes beyond a single gene-phenotype relation. Some of the groundwork for studying phenotypes in a more comprehensive manner has been laid by Piano et al. [4], who used manually curated phenotypes from a single RNAi screen. They described a phenotype as the sum of 45 phenotypic features and coined the term 'phenoclusters' (which we adopt here) to describe groups of such vectorized phenotypes that 'correlate well with sequence-based functional predictions and thus may be useful in predicting functions of uncharacterized genes' [4]. Similarly, a study by van Driel et al. [12] compared human phenotype descriptions and found that grouping of phenotypes reflects biological modules of interacting functionally related genes. Recently, Gaulton et al. [13] developed a computational system to suggest new genes contributing towards a 'complex trait' (i.e. a phenotype). They use ontologies and entity recognition to extract genes and proteins from phenotype descriptions and rank them accordingly to corresponding biological data from online resources. Butte et al. [14] clustered keywords from the Unified Medical Language System (UMLS) annotated to gene expression data and interpret the resulting connection between these terms and the associated genes (termed 'phenome-genome network'). In a study by Raychaudhuri et al. [15], text-mining methods were applied to Medline abstracts dealing with gene function – but not specifically phenotypes – and to assign functional annotation from the Gene Ontology (GO) [16]. Using phenotype data for more than annotation prediction, Eggert et al. [17] compared phenotypes from RNAi as well as chemical genetic screens to find genes responsible for the same cellular phenotype. Thus, they could identify new members of known pathways as well as small molecules with an effect on the same pathway.

All these approaches have in common that they worked either with only very little phenotype data (usually only one data set from one screen) or with a large but very unspecific set of 'phenotypes' (such as all Medline abstracts). In this paper, we go beyond these limitations and present the results of a systematic and large-scale comparison of phenotypes from multiple sources and touching upon many species. We used PhenomicDB [9,10] as one of the largest available collections of phenotype descriptions across species. PhenomicDB aims at the integration of phenotype data in any of the above mentioned senses. Therefore, and due to the lack of standardized vocabulary as explained above, it stores phenotype descriptions mostly as plain text extracted from the various sources. We analyzed these data using text-clustering to group together similar phenotypes, therein creating a novel approach to cluster genes. To validate the biological usefulness of the created 'phenoclusters' (which are a generalization of the 'phenoclusters' as defined by Piano et al.), we examined the relatedness of the genes in a cluster using several independent measures, i.e., protein-protein-interaction (PPi) of associated proteins, functional annotations from the Gene Ontology (GO), and the co-occurrence of pairs of genes known to be responsible for identical phenotypes (so-called 'phenocopies'). We found that 'phenoclusters' have interesting properties: They significantly correlate with the degree of connectedness on the PPi level, phenocopies co-occur significantly more often in the same cluster than in different clusters and they are highly enriched in terms of coherence of their functional annotation. Especially the last observation led us to the hypothesis that genes within a 'phenocluster' have a high chance of sharing gene function, and that we can use 'phenoclusters' for function prediction by predicting the function of badly characterized or un-annotated genes in a cluster from the function of other genes in the same cluster. Using cross-validation, we found that this method yields a recall of up to 16.7% at a precision of 72.6%, which, in our mind, strongly supports our hypothesis. We conclude that 'phenoclusters' are a novel and promising way of finding relationships between genes with high biological coherence.

## Results

We obtained textual description of phenotypes and a reference to their associated gene from the PhenomicDB database. For text mining purposes, the descriptions had to be properly adapted and prepared (stemming, etc.). We use the working term 'phenodoc' in the following to refer to this adjusted form of phenotype description and use 'phenocluster' to refer to a cluster of 'phenodocs'. We clustered the resulting 39,610 'phenodocs' associated to 15,426 genes from 7 different species into 1,000 clusters based on the cosine distance between 'phenodocs' using the k-means algorithm on a vectorized representation of the documents. We studied the resulting groups from a number of perspectives to assess whether or not the grouping itself is biologically reasonable. Finally, we predicted gene function within each cluster and evaluated this method using cross validation. All methods are described in detail in the 'Methods' section.

**Figure 1:**
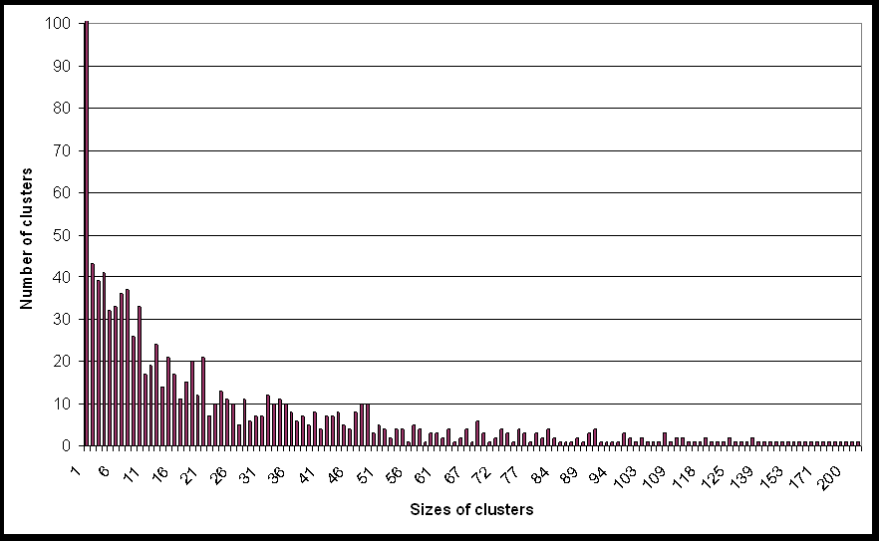
**Distribution of cluster sizes**. The diagram shows the distribution of the number of clusters in different sizes.

**Figure 2:**
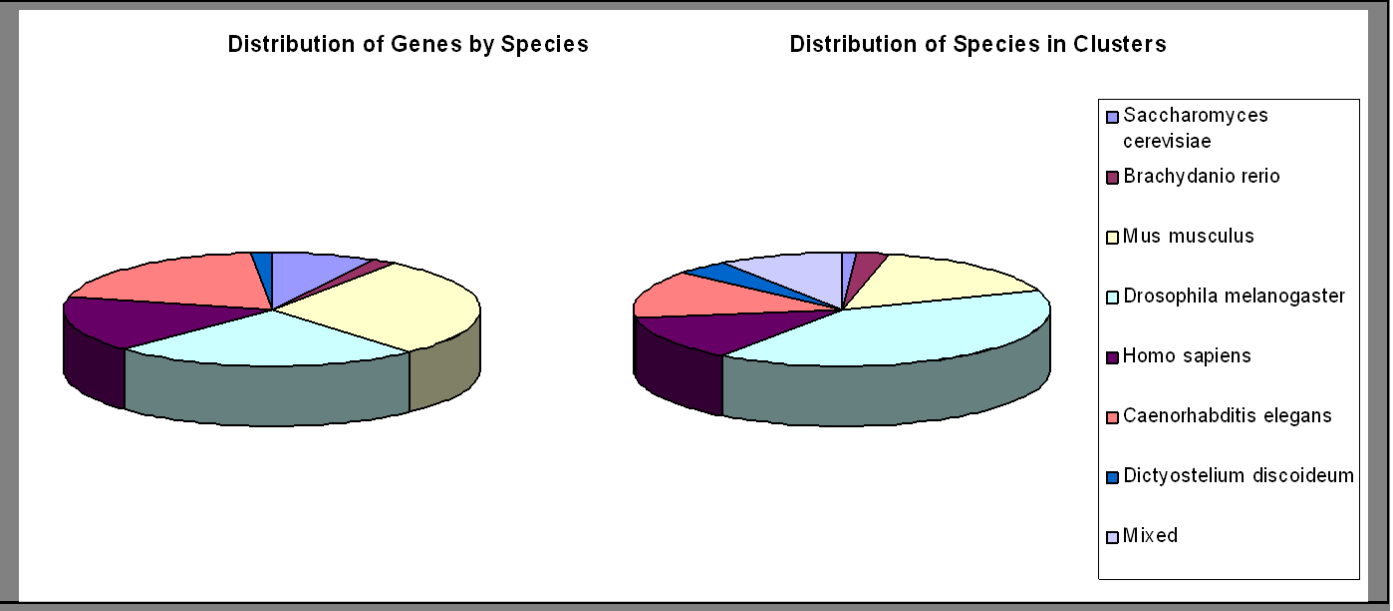
**Cross-species phenotype data distribution**. The left pie chart depicts the distribution of genes by species, i.e. the relative number of genes in our gene set according to species affiliation. The right pie chart shows the distribution of clusters according to single species or 'mixed', if the cluster is made up of genes from more than one species.

Of the 1,000 clusters, 90.4% are single species. Figure 1 shows the distribution of clusters into different sizes. Figure 2 details the distribution of genes by species (independent of the clustering) and the distribution of species in clusters (dependent on the clustering).

### Proteins of genes within a 'phenocluster' intensively interact with each other

To test whether 'phenoclusters' consist of genes with a high chance of being part of a common biological process, we studied whether the proteins of genes within one cluster interact with each other more often than of genes in random control groups. This approach derives from the observation that physically interacting proteins have a higher chance to be part of the same biological process or pathway than non-interacting proteins [18]. To this end, we downloaded protein-protein interactions from the BioGrid database represented by Entrez Gene IDs (see 'Methods'). We then analyzed the degree of interactions among the members of a given 'phenocluster', and compared those figures to random gene groups of similar size.

In 60 clusters (from 1,000) comprising 1,858 genes, all genes interact with at least 75% of the rest of the genes from the same group within at most two intermediates (empirical p-value smaller than 0.05). Thus, those clusters consist of genes which almost build cliques in the protein-protein-interaction network. Such quasi-cliques previously have been associated to functional modules [19]. In another 138 clusters, comprising a total of 4,322 genes, all genes interact with at least 33% of the rest of the genes in each group. We compared these numbers to 200 repetitions of randomly sampled control groups. In this dataset, there is on average only one group reaching the threshold of 75% and two groups reaching the threshold of 33%.

These figures show that clustering of 'phenodocs' results in gene groups whose members much more often interact with each other than expected by chance and thus represent coherent biological knowledge. However, the interaction score of the rest of these clusters is not significantly higher than in the control groups. We shall later exploit this difference to sort clusters based on this score to see whether the prediction of function is improved in highly interacting clusters.

**Figure 3:**
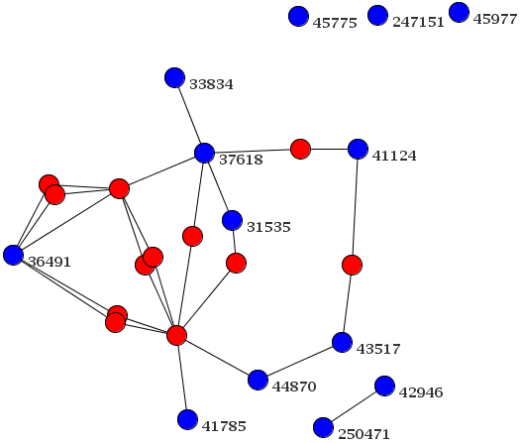
**Protein-Protein interactions derived from one 'phenocluster' and genes lacking phenotype data**. The figure shows an example for interactions between proteins from genes in a 'phenocluster'. Depicted is a network with many genes from the same 'phenocluster' (blue nodes with Entrez Gene IDs) for which associated proteins are connected, while the genes of all proteins that are responsible for these connections are not in our initial set of genes due to lack of substantial phenotype data (red nodes).

**Figure 4:**
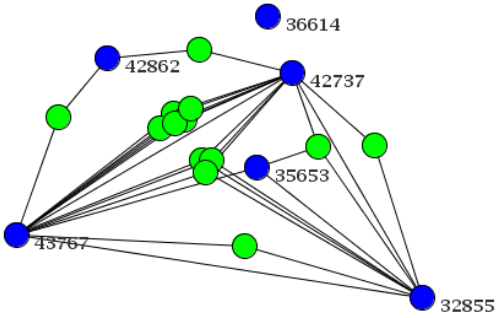
**Protein-Protein interactions of proteins derived from several 'phenoclusters'**. The figure shows an example for interactions between proteins from genes of several 'phenoclusters'. Depicted is a network with many genes from the same 'phenocluster' (blue nodes with Entrez Gene IDs) for which associated proteins are connected. Also, other genes with known phenotypes for which proteins are responsible for some connections are not in the same 'phenocluster' but in the same network (shown as green nodes).

We believe that the large number of those non-interacting clusters is mostly an artefact of the current incompleteness of PPi data sets in BioGrid, with the notable exception of *Saccharomyces cerevisiae*. Therefore, even highly interacting 'phenoclusters' will not necessarily mimic the PPi network due to the diverse nature of phenotypes or a lack of data (both on the PPi and the phenotype side). Figure 3 exemplifies e.g. the lack of phenotype data showing genes from a 'phenocluster' with many connected proteins (blue nodes), where we have added *a-posteriori *and coloured in red those genes having interacting proteins but no phenotype described yet. In contrast, in Figure 4 the clustered blue nodes are again supplemented by nodes from the PPi data. Here, we find nodes added *a-posteriori *with phenotype data (green) that have been clustered elsewhere and one single unconnected node. Both these examples show that phenotype data is only in some way congruent with PPi data (see 'Discussion'). Nevertheless, our 'phenoclusters' give insight into the structure of biological networks and can be used to identify new members in a sub-network not detected by other methods, e.g. the only unconnected node in Figure 4 could be such a case.

### Genes in 'phenoclusters' have coherent GO-annotations

The Gene Ontology (GO) has been widely recognized as the most comprehensive functional classification system and has become a *de facto *international standard for functional annotation and prediction [20-22]. It should be noted here that in PhenomicDB, Gene Ontology terms are associated to the gene descriptions and are not part of our 'phenodocs' (unless by rare coincidence, i.e. when authors had used terms in the free text descriptions that may also occur in GO). Therefore, as a second way of evaluating the biological meaning of 'phenoclusters', we computed the similarity of the GO-terms assigned to the genes of a group (see 'Methods' for calculation and interpretation of the following similarity scores). In the analysis of our 1,000 'phenoclusters', we found 206 clusters containing 1,800 genes with a GO-similarity score ≤ 0.4. For each distinct group size we built 200 control groups from randomly picked genes. Only two control groups reached this threshold by chance. We furthermore computed the correlation of the average GO-similarity with the average phenotype similarity of clusters. The Pearson correlation coefficient *r *was 0.41, indicating a shared variance in both similarity scores, approximately 16% higher than expected by chance.

This shows that phenotype similarity is indicative for a high probability to share GO-annotations between the associated genes. In Table 1 we present an exemplary cluster with a GO-similarity score of 0.9. Of all terms associated with this group, there are 5 terms annotated to 14 out of 17 genes. Due to the homogeneous nature of the annotations, one can hypothesize that the remaining 3 genes should receive the same common annotation as the other 14 genes. We shall build on this idea later when we predict GO-terms in 'phenoclusters'.

**Table 1:** 'Phenocluster' with 17 associated genes with a GO-score of 0.9 in the Biological Process subtree.

Entrez ID	Gene Symbol	Gene name	# annotated GO-process terms	# terms common to at least 50% genes in group	# terms common to at least 75% genes in group
172805	rps-19	Ribosomal Protein, Small subunit 19	5	5	5
174346	eif-3.G	Eukaryotic Initiation Factor	7	4	4
175501	rpl-3	Ribosomal Protein, Large subunit 3	6	6	5
175538	lrs-1	Leucyl tRNA Synthetase	14	6	5
175584	rps-19	Ribosomal Protein, Small subunit 1	7	6	5
175659	rrt1	aRginyl aa-tRNA syntheTase	8	4	4
175796	rpl-23	Ribosomal Protein, Large subunit 23	8	6	5
175901	rps-13	Ribosomal Protein, Small subunit 13	5	5	5
176007	rpl-36	Ribosomal Protein, Large subunit 36	6	6	5
176011	rps-21	Ribosomal Protein, Small subunit 21	6	6	5
176024	prs-1	Prolyl tRNA Synthetase	9	6	5
176071	rpl-9	Ribosomal Protein, Large subunit 9	7	6	5
176097	rpl-35	Ribosomal Protein, Large subunit 35	5	5	5
176146	rpl-21	Ribosomal Protein, Large subunit 21	5	5	5
177583	rps-21	Ribosomal Protein, Small subunit 2	5	5	5
179063	W02F12.5	W02F12.5	8	5	5
189611	Y37B11A.3	Y37B11A.3	2	1	1

Of all terms associated with this group, there are 5 terms annotated to 14 out of 17 genes. Due to the homogeneous nature of the annotations, one can hypothesize that the remaining 3 genes should receive the same common annotation as the other 14 genes.

### Phenocopies co-occur in 'phenoclusters'

A phenocopy is an environmental effect of a single trait (phenotype) that mimics the effect of a trait produced by a gene, which is in this case intact, i.e. wild-type. However, there are also phenocopies independently induced by different genes. In an extensive manual search of Medline literature on phenocopies induced by genes, we have identified 27 of such phenocopies, induced by 57 genes in total (see Additional file 1 for details on the phenocopies and the literature evidence). If our 'phenoclusters' properly reflect phenotype similarity on a biological basis, the genes causing phenocopies should co-occur within the same clusters. Of the 27 phenocopies induced by 57 genes we have retrieved from literature, 25 phenocopies (55 genes) were in our data set. In our 1,000 'phenoclusters', the genes of 13 (54.2%) phenocopies co-occurred in a cluster. In 1,000 random clusters of the same sizes none of those genes co-occurred in any cluster.

### Predicting gene function within 'phenoclusters'

Based on the previous results, we hypothesized that gene function can be predicted based on the association of genes to 'phenoclusters'. If gene groups based on 'phenoclusters' have a coherent GO-annotation, we should be able to predict similar functions in genes from the same cluster (see 'Methods').

In evaluating the correctness of a GO-annotation prediction, one has to consider the structure of the gene ontology. Recall that GO-terms form an ontology, and that terms are connected by IS-A and PART-OF relationships. The simplest case would be to consider a prediction as correct only when it appears exactly as it is in the test data. However, this measure is overly harsh, since terms being a little more general or more specific are also very useful from a biological point-of-view. In the following, we therefore give results for different definitions of 'correctness' of a prediction of a term. In the most stringent case, we consider a term to be correct only if it appears itself in both, test and training set. Thus, predicting a child of a term actually counts negative twice – as a false positive and a false negative. Because this measure is much stricter than that of other studies (see for instance [23]), we also studied how our figures change when we apply a less stringent criterion for 'equality' of GO-terms.

In the following section, we present values for precision and recall of GO term predictions for different subgroups of genes from our 'phenodocs'. Our 'predictions' show the percentage of overlaps between the true annotations of a set of genes (test set) and 'predicted' terms which are derived from a training set (according to the Entrez Gene2GO annotations – see 'Methods' for further details).

### Precision ceiling

To explore the upper limit of 'predictability' of GO-terms based on phenotype clustering using our method (the so-called 'precision ceiling'), we first ran the following experiment. We performed function prediction for all gene groups based on the clustering of the 'phenodocs'. For each group, we computed precision and recall of the predictions. We then selected the 10% highest-scoring clusters sorted by the harmonic mean of recall and precision (so-called F-Measure). Thus, clusters were selected *a-posteriori *based on their performance in prediction. Of course, this measure cannot be extrapolated to the result of a prediction on unknown groups; however, it gives a good estimate on the maximum performance achievable using our data set and our approach. Function prediction from only these groups yielded an average 81.5% precision and 61.2% recall. Considering this as upper limit, we strived for criteria for selecting appropriate gene groups *a-priori*.

### Results for different filter criteria

We defined a number of filters for selecting clusters, based on criteria such as the number of genes they contain, the number of available annotations, and their scores for in-group annotation coherence and in-group connectedness. We defined five different filters which are described in Table 2. We calculated precision and recall of function prediction in all clusters selected by different combinations of those filters, see Table 3 (refer to 'Methods' for details on filters and evaluation). Using the least stringent filter (Filter 1), but the strict criterion for judging the identity of GO terms, the number of clusters was reduced to 856 by filtering all clusters containing less than 3 genes and reduced once more to 295 by filtering all clusters without any descriptive GO-terms (i.e. any Biological Process terms assigned to at least 50% of cluster members). We predicted 345 distinct GO-terms from the Biological Process subtree at a precision of 67.9% and a recall of 23.0%, averaged over all selected clusters.

**Table 2:** Different criteria for filtering clusters for function prediction

	(Filter 1)	(Filter 1 & Filter 2)	(Filter 1 & Filter 3)	(Filter 1 & Filter 4)	(Filter 1 & Filter 5)
# of groups	196	74	53	185	11
# of terms	345	159	102	338	16
# of genes	3213	711	409	2895	320
Precision	67.91%	62.52%	60.52%	67.73%	64.70%
Recall	22.98%	26.16%	19.78%	23.80%	11.21%

In order to push the values for precision and recall towards the precision ceiling, we strived for filter criteria for selecting appropriate gene groups *a-priori*. To achieve this goal, we defined the following filter criteria for our 1,000 'phenoclusters':

Filter 1: Removes groups with less than 3 genes, no GO-terms associated to at least 50% of genes

Filter 2: Removes groups with a GO-similarity score < 0.4

Filter 3: Removes groups with a PPi-connectedness < 33%.

Filter 4: removes all non-single species clusters.

Filter 5: removes all single-species clusters

**Table 3:** Results for different filters applied to gene groups (k = 1,000).

K	500	1,000	2,000	3,000
Single Species cluster	422 (84.4%)	904 (90.4%)	1897 (94.9%)	2894 (96.5%)
# of Phenocopy-Pairs (of 25)	25 (100%)	13 (52%)	12 (48%)	8 (32%)
Cluster w/PT-Sim = 0.4	92 (18.4%)	293 (29.3%)	526 (26.3%)	810 (40.5%)
# Genes	3221	5886	6379	6878
Cluster w/GO-Sim = 0.4	51 (10.2%)	206 (20.6%)	522 (26.1%)	921 (46.1%)

Precision and recall values of function prediction in all clusters and with varying k selected by different combinations of the filters defined in Table 2.

Relaxing the criteria for GO-term identity, now allowing for a single deviation towards the root (i.e., a predicted term is considered correct if it exactly matches a removed term or if it matches a parent of the removed term) resulted in an average 75.6% precision and 28.7% recall (191 unique terms for 2,686 genes in 279 groups). Allowing one more step towards the root, we predicted 151 unique terms with 76.3% precision and 30.7% recall.

If we used for function prediction only those clusters that pass Filter 1 and that show an average GO-similarity ≤ 0.4 (Filter 2), the averaged precision dropped slightly to 62.5% and recall increased to 26.2% (74 groups, 711 genes and 159 predicted distinct GO-process terms). This drop in precision and increase in recall is due to the increasing number of predictions made per gene and group and is explained in more detail in the following sections. Applying again a less stringent criterion for identity of GO-terms as explained above, we derived an average 75.3% precision and 31.7% recall in the first step towards the root (91 unique terms for 612 genes in 80 groups). When we selected only those clusters containing genes from only one species (Filter 4), the values for precision and recall stayed roughly the same. This was expected as 90% of all clusters met to this condition (see 'Discussion'). The values for precision dropped slightly and for recall quite dramatically when we used only cross-species clusters (Filter 5).

To our surprise, average precision and recall dropped (to 60.5% and 19.8% respectively; 53 groups, 409 genes and 102 GO-terms) when we used only those clusters that show a PPi-connectivity of at least 33% (Filter 3). In a recent study [24] it was reported that 35% of interactions occur between proteins with no common functional annotation. We believe that lack of common functional annotations in relatively small groups of immediate neighbours in PPi-networks explain our surprising drop in precision and recall when using only these groups. Nevertheless, both enrichment in pairwise interactions and common GO-terms show the high biological coherence of 'phenoclusters'. We conclude that despite some shortcomings in the data, 'phenoclusters' appear to be another suitable source functional annotation prediction.

### Selecting gene groups from PPi-cliques

To see whether our prediction method using 'phenoclusters' exceeds the use of another non-random gene selection method; we grouped our 13,068 initial genes based on direct pair-wise interaction. We found 2,875 groups in which each gene interacts with each other (i.e. cliques in the PPi graph). Applying Filter 1 on this data set, we derived 720 groups resulting in 3,692 predictions with a precision of 56.4% and 32.3% recall. Thus, the precision of this approach (which is similar to the method applied in [19]) was about 10–20% less precise than our method of clustering genes based on 'phenodoc' similarity.

### Clustering phenotypes with different values of k

K-Means is a clustering method that requires the *a-priori *determination of the number of clusters k. Typically, to assess cluster quality internal and external measures are evaluated [25,26]. External measures, however, as e.g. a comparison with a gold standard, cannot be applied here due to the lack of a gold standard for clustered 'phenodocs'. As internal measure for cluster quality, we sought to gain insight how the data structure changes by choosing different values for k, ranging from 500 to 3,000 (Table 4). The results show a number of interesting facts. Firstly, the average number of genes per cluster clearly decreased with increasing k. However, the percentage of clusters that comply with Filter 1 in Table 2 stayed roughly the same. Although those clusters on average contained fewer genes, the number of predicted annotations and affected genes increased considerably with increasing k. This indicates that the top clusters – selected by Filter 1 – become more homogeneous with increasing k, as more clusters have more terms which are annotated to more than 50% of their members. Partly, this is also a statistical effect of the decreasing cluster sizes which naturally lead to more homogeneous groups. At the same time, the precision drops slightly with increasing k while recall increases considerably. This means, that more predictions come along with more errors, but the ratio of errors to the overall amount of predictions decreases. Another effect is that in smaller clusters, there is usually only a single gene left in the test set. The increasing recall shows that more terms from the test set are descriptive in the training set, but the decreasing precision means that the number of terms associated with a single gene cannot compensate for the number of suggestions derived from the training set.

**Table 4:** The distribution of clusters with their characteristics given different values for k (the number of clusters) from 500 to 3,000.

K	500	1,000	2,000	3,000
Single Species cluster	422 (84.4%)	904 (90.4%)	1897 (94.9%)	2894 (96.5%)
# of Phenocopy-Pairs (of 25)	25 (100%)	13 (52%)	12 (48%)	8 (32%)
Cluster w/PT-Sim ≥ 0.4	92 (18.4%)	293 (29.3%)	526 (26.3%)	810 (40.5%)
# Genes	3221	5886	6379	6878
Cluster w/GO-Sim ≥ 0.4	51 (10.2%)	206 (20.6%)	522 (26.1%)	921 (46.1%)
Correlation GO-Sim vs PT-SIM	0.53	0.41	0.37	0.28
# Genes	863	1800	2392	3065
Cluster w/PPi ≥ 75%	21 (4.2%)	60 (6.0%)	174 (8.7%)	305 (10.2%)
# Genes	1497	1858	2335	2702
Cluster w/PPi ≥ 33%	63 (12.6%)	138 (13.8%)	286 (14.3%)	413 (13.8%)
# Genes	3890	4322	4965	4996
Cluster for GO-Predictions	90 (18%)	196 (19.6%)	393 (19.7%)	611 (20.4%)
# Genes	2820	3213	4145	4546
# Terms	142	345	730	1226
Precision	72.55%	67.91%	63.40%	60.31%
Recall	16.73%	22.98%	25.63%	28.32%
Avg. Genes/Cluster	54	29	16	11

As internal measure for cluster quality we sought to gain insight how the data structure changes by choosing different values for k, ranging from 500 to 3,000. Here, Filter 1 has been applied for GO-predictions. For details, see text.

While the correlation between GO-similarity and phenotype similarity drops significantly for increasing k, the percentage of single-species clusters increases. This is an indication that the homogeneity within clusters mentioned above shifts from a functional to a methodical, i.e. a descriptive homogeneity owned by the fact that similar vocabulary – from the same species – yields less variance than similar function.

Thus, k is an important parameter to balance the trade-offs between precision, recall and number of predictions. One can either choose a small k-value, resulting in few high quality predictions, or a larger k-value, resulting in a much larger number of less accurate predictions. Clearly, the choice of the k-value depends on the concrete application. As our goal was the best precision with acceptable recall, we found k = 1,000 most suited, although a large k (k = 3,000) resulting in many small clusters yields the best technical solution with an F-Measure of 0.385 (precision = 60.3% and recall = 28.3%).

## Discussion

### Using phenotypic data

For a long time, phenotypes have been regarded solely as indicators for changes in genotypes or diseases. The ability to interfere with the genetic component in a systematic manner, e.g. by gene knock-out or RNA interference [6,7], has raised the importance of phenotypes as a tool to understand biological processes on the molecular level. Even though whole-genome RNAi screens have created large amounts of phenotype data, many of them publicly available, very few attempts have been reported to systematically analyze these data beyond single gene effects. It is noteworthy that even 5 years after the availability of RNAi screens for mammals and many calls for standardization of data types [27,28] and analysis methods in phenomics, still, such data is poorly organized and difficult to access. Only recently, the Eumorphia project has released standard operating procedures for phenotype screening in the mouse and has created PhenoStat, a tool for visualization and systematic statistical analysis of standardized phenotype data [29,30].

Our approach contributes a new method to cluster genes based on their functional relationships on a high level. We have shown that such clusters can be used to identify and predict gene function and interaction, and that they have a high biological coherence.

### Examples for gene groups

The evaluation of our prediction method has the fundamental drawback that only existing annotations are considered as correct. However, it is well known that GO-annotations are highly incomplete for virtually all species. Thus, we have a considerable chance for false predictions that actually may be the most interesting ones from a biological point of view. False positive predictions potentially represent new functional insights, e.g. when a gene not yet annotated with a particular function is found in a cluster with a strong consensus annotation for that function. In the following, we discuss the biological nature of exemplary 'phenoclusters' to show their biological significance beyond pure precision values.

### Example 1: Odorant receptors from *Drosophila melanogaster*

One of our clusters consisting of 25 genes shows a high consensus annotation for the three GO-terms *G-protein coupled receptor protein signaling pathway *(GO:0007186), *sensory perception of smell *(GO:0007608) and *cell-cell signaling *(GO:0007267). This group contains 24 genes from the *Drosophila melanogaster *odorant receptor (*DOR*) group and one other gene. This other gene is called *myospheroid *(*mys*). It is in several ways a very interesting group:

Firstly, all 24 genes are antennal *DOR *genes, a physiological region of *Drosophila melanogaster *in which a total of 32 *DOR *genes are located [31]. The other 8 antennal *DOR *genes not found in this group (*Or13a*, *Or22b*, *Or33a*, *Or42b*, *Or56a*, *Or69a*, *Or69b *and *Or83c*) are not in our initial list of 15,426 genes, likely due to a lack of substantial phenotype description. 13 of the 24 genes are annotated with all three GO-terms from the consensus annotation in that cluster, the other 11 genes are only annotated with GO-term *sensory perception of smell *(GO:0007608). The other interesting aspect is the occurrence of *mys*, neither a gene from the DOR group, nor annotated with any of the three GO-terms, but instead with 19 other terms, among them *signal transduction*, *axon guidance*, *calcium-dependent cell-cell adhesion*, *calcium-dependent cell-matrix adhesion*, *cell adhesion*, *cell migration *and *cell-matrix adhesion*. Even though it has been previously suggested that *mys *is a candidate gene for *Drosophila *olfactory associative learning [32], only recently, in a publication not yet included in PhenomicDB, a link between the *Drosophila *olfactory system and *mys *has been reported. Bhandari et al. have shown that expression of *mys*-RNAi transgenes in the antennae, antennal lobes, and mushroom bodies disrupted olfactory behaviour, confirming that *mys *is important for the development and function of the *Drosophila *olfactory system [33].

It is not clear why some of the antennal receptors are not annotated with the two GO-terms *G-protein coupled receptor protein signaling pathway *and *cell-cell signaling*. After all, all are clearly odorant receptor proteins consisting of seven transmembrane domains, and transduce odour recognition into neuronal activation through G-protein-coupled second messenger signalling pathways [34]. In our analysis of GO-annotations, these genes represent false false positive results, i.e., the annotation that has been predicted is in fact true but missing, thus reducing the precision of our prediction.

Another important lesson that can be learned from this example is the dependence of phenotypic similarity on complete and well-structured phenotypic data. Even though one study reports on both antennal receptors *Or22a *and *Or22b *(which are co-expressed in the ab3A antennal neuron and share 78% amino acid identity, separated by an intergenic region of only 650 base pairs [35]), our gene group only includes *OR22a*, simply due to missing phenotypic information about the other gene.

### Example 2: 8 *Drosophila melanogaster *genes

In another group, yielding very high average pair-wise phenotype- and GO-similarities, most genes are associated with the two GO-terms *cellularization *(GO:0007349) and *pole cell formation *(GO:0007279). This group consists of 8 *Drosophila melanogaster *genes, including 6 genes from the *mat(2)syn *(maternal effect syncytial blastoderm arrest) family, as well as the *indirect flight muscle gene RU2 *(*ifm(2)RU2*) and a gene called *presto*. *Presto *and the genes from the *mat(2)syn *group are both associated with deficiencies on the second *Drosophila melanogaster *chromosome and are part of closely related maternal effect loci that cause defects before cellularization of the blastoderm embryo [36]. During these stages of nuclear division, the embryo is called a syncytial blastoderm, meaning that all the cleavage nuclei are contained within a common cytoplasm, including cleavages that form the two cell types of early development (pole cell and blastoderm cell) [37].

Seven genes are annotated with both GO-terms, while *ifm(2)RU2 *is not annotated with any of those terms. Instead, it is annotated with the term *muscle development *(GO:0007517). Still, all phenotypes in the cluster show a high similarity and this may indicate that the genes are commonly regulated or that they are part of one developmental process. The development of the indirect flight muscle has been closely associated with the *myosin heavy chain *gene (*MHC*) [38]. Since *ifm(2)RU2 *and *MHC *are found on very close-by loci, they have been studied together in an ethyl methanesulfonate mutagenesis screen [39], where mutated *MHC *and *ifm(2)RU2 *have been found to act together, enhancing muscle disorganization compared to their respective heterozygous phenotypes.

Another gene, the *95F unconventional myosin *gene (*95F MHC*) is shown to be required for proper organization of the *Drosophila melanogaster *syncytial blastoderm [40]. Compared to *MHC*, this gene shows a high degree of conservation in the ATP-binding and actin-binding regions, and SH2, one of the two reactive thiols (SH1 and SH2) found in many muscle *MHC *heads is also present in *95F MHC*. The amino-terminal two thirds of the protein comprise a head domain that is 29–33% identical (60–65% similar) to other myosin heads, and contains ATP-binding, actin-binding and calmodulin/myosin light chain-binding motifs [41].

When looking only at GO-annotations, this gene is a true false positive result, i.e., its available annotation is in line with the biology but not part of the consensus annotation for the rest of the genes from this cluster, thus the prediction is wrong. Even though there is not yet proof for an immediate interaction between *MHC *and *95F MHC*, these genes are very similar to one another. A relationship between *MHC *and *ifm(2)RU2 *has already been suggested and we have found further indications that those genes are responsible for similar phenotypes (and the term 'myosin' does not occur in any of the phenotype descriptions). We have therefore reasons to believe that there are some undisclosed functional links between *95F MHC*, *MHC*, *ifm(2)RU2 *and at least some *mat(2)syn *gene family members. We provide these very interesting findings to be tested biologically.

### Cross-species clustering

As stated, more than 90% of clusters contain only genes from a single species. However, as can be seen in Figure 2, the distribution of species in clusters differs greatly from the distribution of species in the gene set. For example, although almost a third (28.7%) of the genes from our set are mouse genes, they can be found in just 16% of the clusters, while less than a quarter (23.9%) of fly genes make up 39.9% of the clusters. This is a clear indication towards the heterogeneity within the different data sets. On the other hand, the very strong tendency of genes to fall into species-specific clusters shows that the terminology that is used to describe a phenotype strongly depends on the species, and thus on the community of researchers studying it. A prominent example of community-specific annotation is the Mammalian Phenotype Ontology [43] used to annotate many phenotypes for mouse genes but, of course, not for fly genes. This separation of terms is only partly justified, as many phenotype-effects are equal across-species. However, until now, no common terminology for describing phenotypes in different species has been established. We believe that such a unified ontology would open the door to more powerful ways of analyzing phenotypes, in the same manner as the establishment of the Gene Ontology has opened the door for many new approaches to analyze biological knowledge.

## Conclusion

We have shown that a great deal of the heterogeneous nature of phenotype data can be overcome by using text-mining. Within one framework, we systematically analyzed textual descriptions of clinical diseases, naturally occurring mutants, RNAi screens, gene knock-out experiments, and many others. Using clustering, a reasonable fraction of the associated genes can be grouped into biologically meaningful categories. Grouping genes based on certain properties is a powerful tool that has often been applied for function prediction before, using criteria such as participation in the same pathway [42,43], participation in PPi cliques [19], or mentioning in the same Medline abstracts [15] – but not on phenotypes. We believe (and have shown) that this is an important new approach, as phenotypes, e.g. in contrast to interaction data, yield more information on the high diversity of biological meaning that is innate to any gene. It is, in fact, the intrinsic nature of phenotypes to visibly reflect genetic activity. Thus, phenotype data has the potential to be more useful for functional studies than most other types of data.

A much larger fraction of our clusters are more homogeneous with respect to pair-wise interactions, GO-annotations and re-occurrence of phenocopies than expected by chance. The remaining part of the 'phenodocs' do also cluster, but probably not driven by biology but by spurious effects of the data set itself and the clustering methods we used; an effect typical of high-throughput methods (e.g. gene expression). We showed that 'phenoclusters' can be used to infer gene functions for poorly annotated genes with high precision and reasonable recall. In a recent survey, Pandey et al. [21] have collected success rates for different approaches, like sequence clustering, to protein function prediction using GO. These approaches show precision values between 50% and 74%, i.e. from slightly below to slightly above our values (note that some of the methods applied in these works are not directly comparable to our approach). In the light of this survey, large-scale phenotype clustering as carried out here should be considered as a novel tool to predict function with highly competitive results.

## Methods

### Phenotype Data

We obtained all phenotype data from PhenomicDB [9,10], a cross-species genotype/phenotype database integrating data from the Online Mendelian Inheritance in Man database (OMIM), the Mouse Genome Database (MGD), WormBase, FlyBase, the Comprehensive Yeast Genome Database (CYGD), the Zebrafish Information Network (ZFIN), and the MIPS Arabidopsis thaliana database (MAtDB).

Of 428,150 phenotype entries from PhenomicDB (version 2.1, released October 2006), 411,102 entries are directly associated to at least one gene; only those were considered for our study. For each entry, we collected its Entrez Gene ID and the available text from all associated phenotype entries using the PhenomicDB fields 'names', 'descriptions', 'keywords' and 'references'. We removed all phenotypes with less than 250 characters, as we expect them to be too short to deliver reasonable results in textual comparison and clustering. We stemmed all words using the stemming algorithm from the doc2mat package supplied with the clustering toolkit CLUTO v2.1.1 by Zhao and Karypis [44]. We also removed so-called 'stop-words', which are words of such high frequency that they will not add to the distinctiveness of any feature vector. These stop-words comprise the 200 most common words from the English language and from Medline. All texts were then concatenated into a single string which we call phenotype document or 'phenodoc'. For this study, we also removed the very small number (511 of 428,150) of complex phenotypes as linked to more than one gene. Since PhenomicDB is not normalized in respect to replicate phenotype entries, we did not filter for those associated with different genes (see Additional file 2 for some figures about the phenotypes and 'phenodocs'). We thus obtained a data set of 39,610 'phenodocs' associated with 15,426 genes from 7 different species, i.e. *Brachydanio rerio*, *Caenorhabditis elegans*, *Dictyostelium discoideum*, *Drosophila melanogaster*, *Homo sapiens*, *Mus musculus *and *Saccharomyces cerevisiae*.

This strong data reduction (~9.25% of all phenotypes in PhenomicDB) is due to the large amount of extremely short phenotype description like 'embryonic lethality' created e.g. in high-throughput RNAi screens.

### GO-terms

We obtained all GO-terms from the file 'gene_ontology_edit.obo', format-version 1.2 released August 2007, cvs-version revision 5.461, from the website of the Gene Ontology project [16]. Associations between Gene IDs and GO-term IDs were extracted from the file 'gene2go.gz' from Entrez Gene [45].

### PPi data

We evaluated 'phenoclusters' based on the number of PPi between their associated genes. We obtained PPi data from files BIOGRID-ALL-2.0.33.tab.zip and BIOGRID-IDENTIFIERS-2.0.33.tab.zip from the BioGrid website [46], October 2007. These files contain a list of Entrez Gene identifiers, each associated with all directly interacting Entrez Gene identifiers.

### Clustering 'phenodocs'

We clustered all 'phenodocs' into different numbers of clusters (see the 'Results' section and 'Discussion' for considerations regarding the number of clusters) using the *vcluster *algorithm from CLUTO v2.1.1 by Zhao and Karypis [44], available at [47]. *Vcluster *is a scalable implementation of the well known K-means algorithm for textual data. For calculating 'phenodoc' similarity scores, all 'phenodocs' were transformed into a vector representation with TFIDF scores for each word (TF = 'term frequency', IDF = 'inverse document frequency', TFIDF = TF * IDF) as suggested by Steinbach et al. [48]. Each 'phenodoc' thus consists of the same number of features, some of which may be zero, where the word representing the feature is not found in the 'phenodoc' (see Additional file 2 for information on these features). As criterion function to assign 'phenodoc' vectors to clusters we applied the I_2 _function which is used in most vector-space variants of the K-means algorithm [49]. In addition, *vcluster *needs a measure to judge the similarity of two given 'phenodoc' vectors. The most popular one probably is the Euclidian distance measure, but for clustering texts, the cosine measure is generally considered as more suitable [48]. In a feature space defined only by terms, the direction of a vector may be interpreted as 'theme' of a text. Thus, we determined the similarity of two vectors by their normalized dot product after normalizing vector length. The resulting scores range from 0 (no similarity) to 1 (equality) and represent the degree of thematic relatedness, i.e. the abundance of the same words in both texts.

### Gene similarity based on GO-annotation

We computed the coherence of functional annotation of a group of genes in order to judge the quality of gene groups derived from clusters of 'phenodocs'. This can be accomplished effectively by regarding the similarity of two genes as the similarity of their GO-annotations. There are a number of similarity measures for pairs of GO-terms, many of which have been reviewed and compared by Guo et al. [18]. For calculating the similarity a pair of GO-terms, we use the similarity measure proposed by Lin [18,50]. The resulting score ranges between 0 (when the two terms are connected only via the root) and 1 (when the terms are equal).

Equally to the similarity of GO terms, there have been many suggestions for calculating the similarity of two genes based on their GO-annotations [50-53]. Lord et al. [52] suggested taking the average of all pairwise term similarities from the two sets of terms. In contrast, Wang et al. [54] suggested to take the maximum similarity of all term pairs. The approach by Wang et al. considers only a single pair of terms, i.e. the one pair yielding the maximum similarity, whereas the approach by Lord et al. considers all pairs of terms, even when such a pair has nothing in common, e.g. when the genes are involved in distinct processes. In our view, both measures are unsatisfactory. Furthermore, both measures do not normalize for the number of annotations, i.e. when one set of terms is larger than the other set of terms.

Therefore, we used a slight variation of these approaches. Instead of considering either only one pair or all pairs of terms from either set, we use the k best scoring term pairs with k being the size of the smaller term group. Each term of the smaller set contributes exactly its best score. As an example, consider the case that one gene has ten associated GO-terms associated and another gene has only three GO-terms. We then consider as GO-similarity of the two genes the average of the three best similarity scores from three terms of the smaller set respectively. We believe that this score better reflects the nature of GO-annotations, where genes are unequally well annotated or genes only partially share functions with each other. Our suggested score also ranges between 0, where all terms connected only via the root, and 1 where the k best terms are equal. Any score in-between accounts for a linear degree of similarity between the k best-scoring term pairs, e.g. that a score of 0.5 accounts approximately for one half of k best-scoring terms being equal (or a slightly larger proportion of terms being only one step apart in the hierarchy), etc.

### Correlation between GO-similarity and 'phenodoc' similarity

For each gene pair from a 'phenocluster', two measures can thus be calculated: the GO-similarity score and the average pair-wise similarities of 'phenodocs' associated with the genes. Calculation of each of these measures has been explained above.

From these measures, the average cluster GO-similarity and 'phenodoc' similarity can be computed, resulting in two functional measures of which we can calculate the degree of correlation. We use the commonly known Pearson correlation coefficient *r *(ranging from *r *= -1.0, with perfect inverse linear correlation, over *r *= 0.0, no correlation, to *r *= 1.0, perfect linear correlation).

### Comparison of similarity scores of phenotypes associated with the same gene

We computed the pair-wise similarities of all 'phenodocs' associated to the same gene. The similarity scores were computed by first assigning to each 'phenodoc' a vector of TFIDF-scores based on the words within each document and then calculating the cosine distance between all vectors of 'phenodocs' directly associated to the same gene in PhenomicDB. For the control groups, we randomly picked groups of 'phenodocs' of identical sizes. All results were averaged over 200 runs.

### Evaluation of function prediction

To estimate precision and recall of our approach to function predictions, we considered all 856 clusters with at least three associated genes. We assume GO-terms to be descriptive for a cluster if common to at least 50% of its members and filtered clusters with no descriptive terms. We randomly partitioned each of the resulting 295 clusters comprising 4,438 genes into a training set of 90% of its genes and a test set of at least one gene or 10% of genes, respectively. The descriptive terms of the training set were 'predicted' as new annotations to all genes in the test set of the same cluster. We then compared these predictions to the real annotation of the test genes to judge prediction correctness. This procedure was repeated 200 times (with different training/test sets) and averaged precision and recall of the suggested terms was computed. As empirical threshold (p-value smaller than 0.05), we used randomly populated gene groups of equal size. For 99 groups, we could not make a significant prediction below the given p-value. Leaving us 196 groups for which a significant prediction could be made (see Table 2).

## Authors' contributions

PG: carried out the statistical analyses and programming and contributed to the manuscript; BW: provided the phenotype data, conceived parts of the study and contributed to the manuscript; HDP: was the General Manager, contributed the funding and contributed to the manuscript; UL: conceived most of the statistical tests of this study, and drafted and contributed to the manuscript. All authors read and approved the final manuscript.

## Supplementary Material

Additional file 1 Listing of Entrez Gene IDs of the genes which are phenocopies and PubMedIDs of literature giving evidence to this. In this file, the 27 phenocopies we have identified from literature are listed, including the Entrez Gene IDs of each gene which is a phenocopy and the PubMed ID of the evidence for why exactly those genes are said to be phenocopies. Click here for file

Additional file 2 Figures on phenotypes and 'phenodocs'. This file contains some basic information on word and character sizes of our phenotypes and the number and sizes of features in the resulting 'phenodocs'. Click here for file
