# Supplementary material for: Mining phenotypes for gene function prediction
Source: BMC Bioinformatics. 2008 Mar 3;9:136. doi: 10.1186/1471-2105-9-136 (PMC2311305; doi:10.1186/1471-2105-9-136)
Supplement: Additional file 1 — Listing of Entrez Gene IDs of the genes which are phenocopies and PubMedIDs of literature giving evidence to this. In this file, the 27 phenocopies we have identified from literature are listed, including the Entrez Gene IDs of each gene which is a phenocopy and the PubMed ID of the evidence for why exactly those genes are said to be phenocopies. [file 1471-2105-9-136-S1.pdf]

Listing of and evidence for phenocopies

Number	GeneIDs	PubMedIDs
1	38879,36538	16129829
2	42445,31293	12049762
3	15460,22337	11564167
4	16835,100017	15728179,17200716,14717060,16343504
5	18516,27140	15466398
6	36775,43916	11877391
7	41885,39844	17353360
8	11491,13649	16079154
9	19378,15426	15753214
10	44279,34009	11029007
11	35197,41363	16774999
12	3346167,31816	16862128
13	12802,14678	16924491
14	856580,850675	17553781
15	13433,20937	16980612
16	13711,19116	15650748
17	14179,14182	15221377
18	14460,14461,22761	12077323
19	15111,22160	16237669
20	17319,15251	17142669
21	16449,18129	12496659,15509774
22	4000,10269	16079796
23	17283,214162	15199122
24	19164,19165,18128,18129	12424225,15525534
25	17125,55994	15899870
26	14950,74585	15998642
27	69581,22408	11459829
