# Supplementary material for: Mining phenotypes for gene function prediction
Source: BMC Bioinformatics. 2008 Mar 3;9:136. doi: 10.1186/1471-2105-9-136 (PMC2311305; doi:10.1186/1471-2105-9-136)
Supplement: Additional file 2 — Figures on phenotypes and 'phenodocs'. This file contains some basic information on word and character sizes of our phenotypes and the number and sizes of features in the resulting 'phenodocs'. [file 1471-2105-9-136-S2.pdf]

Figures on phenotypes and phenodocs

Number of phenotypes/phenodocs: 39,610

Phenotypes:
Words:
Avg: 169.99
Min: 27
Max: 985

Characters (excluding whitespaces):
Avg: 1135.29
Min: 251
Max: 6528

Number of unique words: 113283

Phenodocs:
Features:
Avg: 67.87
Min: 12
Max: 364

Number of unique features: 73188
